# Immobilization of *Aspergillus oryzae* tyrosine hydroxylase on ZnO nanocrystals for improved stability and catalytic efficiency towards L-dopa production

**DOI:** 10.1038/s41598-023-50198-x

**Published:** 2023-12-18

**Authors:** Ansa Khalid, Sikander Ali, Rukhma, Muhammad Jahangeer, Abid Sarwar, Rubina Nelofer, Tariq Aziz, Metab Alharbi, Abdullah F. Alasmari, Thamer H. Albekairi

**Affiliations:** 1 Institute of Industrial Biotechnology, Government College University, Lahore, Pakistan; 2 Food and Biotechnology Research Center, PCSIR Laboratories Complex Ferozpur Road, Lahore, Pakistan; 3 Laboratory of Animal Health, Food Hygiene, and Quality, Department of Agriculture, University of Ioannina, 471 32 Arta, Greece; 4 Department of Pharmacology and Toxicology, College of Pharmacy, King Saud University, P.O. Box 2455, 11451 Riyadh, Saudi Arabia

**Keywords:** Biochemistry, Biotechnology, Chemical biology, Microbiology, Molecular biology

## Abstract

The current study focuses on the submerged fermentation of tyrosine hydroxylase (TH) from *Aspergillus oryzae* IIB-9 and its immobilization on zinc oxide nanocrystals (ZnO-NPs) for increased L-dopa production. The volume of Vogel’s medium (75 ml), period of incubation (72 h), initial pH (5.5), and size of inoculum (1.5 ml) were optimal for maximum TH activity. The watch glass-dried (WG) and filter paper-dried (FP) ZnO-NPs were prepared and characterized using analytical techniques. The UV–Vis spectra revealed 295 and 285 nm absorption peaks for WG-ZnO-NPs and FP-ZnO-NPs dispersed in isopropanol. X-ray diffraction analysis confirmed the crystalline nature of ZnO-NPs. FTIR spectra band from 740 to 648.1/cm and 735.8/cm to 650.1/cm showed the stretching vibrations of WG-ZnO-NPs and FP-ZnO-NPs, respectively. The particle size of ZnO-NPs observed by scanning electron microscopy (SEM) images was between 130 and 170 nm. Furthermore, the stability of immobilized TH on ZnO-NPs was determined by varying the incubation period (10 min for WG-NPs and 15 min for FP-NPs) and temperature (45 °C and 30 °C for WG and FP-NPs, respectively). Incubating enzymes with various copper, iron, manganese, and zinc salts studied the catalytic efficiency of TH. Immobilization of TH on ZnO-NPs resulted in an 11.05-fold increase in TH activity, thus enhancing stability and catalytic efficiency.

## Introduction

**Figure 1:**
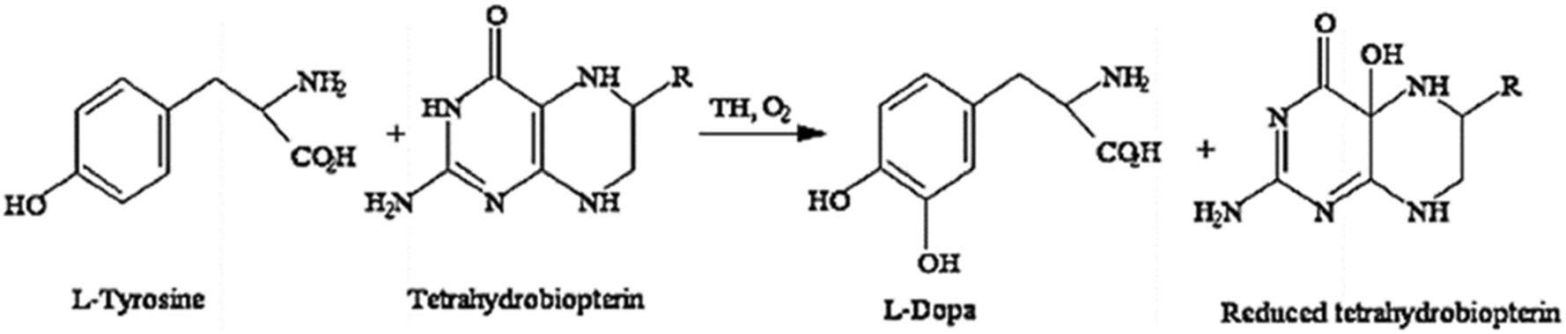
The biotransformation of L-tyrosine into L-dopa by TH enzyme in the presence of tetrahydrobiopterin and O_2_.

Tyrosine hydroxylase (TH), a crucial enzyme involved in the synthesis of L-dopa, is unstable and has a limited catalytic efficiency, which provide challenges to the pharmaceutical industry's efforts to produce L-dopa enzymatically for the treatment of Parkinson's disease and other neurodegenerative disorders. To enhance the stability and catalytic effectiveness of the enzyme and hence improve the L-dopa synthesis process, TH was immobilized on ZnO nanocrystals to overcome this issue. Tyrosine hydroxylase (TH, *E.C. 1.14.16.2*) is a rate-limiting enzyme for producing L-dopa from L-tyrosine^1^, whereas tyrosinase (*E.C. 1.14.18.1*), a copper-containing oxygenase, stimulates the *o*-hydroxylation of tyrosine to 3,4-dihydroxyphenylalanine or dopa^2^. All the dopamine-producing cells produce the TH enzyme, thus ultimately stimulating the synthesis of L-dopa^3^. L-Dopa is a precursor for dopamine and for some other essential neurotransmitters, epinephrine (adrenaline) and nor-epinephrine (nor-adrenaline)^4^. A non-heme ferrous iron (Fe^2+^) and 6R-tetrahydrobiopterin (BH_4_) act as a coenzyme for the TH enzyme. Moreover, molecular oxygen (O_2_) acts as a supplemental substrate to catalyze the reaction undergone by the TH enzyme. Figure 1 represents the biotransformation of L-tyrosine into L-dopa by the TH enzyme in the presence of tetrahydrobiopterin and O_2_^5^. A plethora of sources for TH production has been reported from plants, animals, and insects. In microorganisms, such as *Aspergillus chevalieri* IF04086*, A. oryzae* IAM2625^6^, *A. niger* ^7^*, Citrobacter freundii* ^8^*, *and *Fusobacterium nucleatum* have been used for the production of TH. TH activity, however, is generally very low in microorganisms. Thus, the present work was undertaken to explore *Aspergillus oryzae* as a promising microorganism for producing intracellular TH enzyme under submerged fermentation.

L-dopa’s costly production and extraordinary industrial significance have inspired researchers to explore an alternative approach to biosynthesize L-dopa. The synthesis of L-dopa from the TH enzyme of *A. oryzae* is an economical and feasible method for the production of intracellular TH because of its robust pathways for protein secretion, high capacity for protein synthesis, and adaptability to utilize a variety of substrates, make *A. oryzae* an ideal candidate for producing intracellular enzyme in industrial applications. However, for the cost-effective and large-scale production of TH enzyme and biosynthesis of L-dopa, Vogel’s medium is a convenient medium for the growth of *A. oryzae* ^9^. Certain parameters such as temperature, pH, incubation time, and inoculum size have significant impact on the biosynthesis of the TH enzyme^10^. Optimizing these factors can enhance TH activity, as L-Dopa is an unstable compound that is readily oxidized into free radicals in the presence of light or oxygen. It can be made stable instantly after production by immobilizing the TH enzyme onto an insoluble support matrix. To overcome this problem, the TH enzyme should be made more stable, efficient, and reusable for a more extended period.

Several approaches have been utilized to enhance stability and catalytic efficiency of enzymes, but immobilizing the enzyme on a nanosupport is a better-opted technique. Recently, nanotechnology advancements have intensified nanocrystals' use as microbial enzyme carriers^11^. Nanoparticles (NPs) have gained significant importance due to their considerable demands in several disciplines, i.e., therapeutic, medicinal delivery, etc. In previous studies, gold NPs and porous silicon NPs were used to immobilize the TH enzyme^12^. Amongst various nanosupports, ZnO nanocrystals have emerged as an excellent support for the immobilization of TH because of their extraordinary corporeal and chemical characteristics^13^. ZnO nanocrystals, being biocompatible, are suitable for the immobilization of enzymes without significantly altering their structure or activity. This feature makes them suitable for maintaining the functionality and integrity of the immobilized TH. Previous studies have successfully demonstrated the use of ZnO-nanocrystals to immobilize various enzymes, but TH enzyme immobilization on ZnO nanosupports has not been reported yet. Therefore, this study aimed at immobilizing tyrosine hydroxylase produced from *A. oryzae* on ZnO-nanocrystals to provide new approaches for improved stability and catalytic efficiency of TH enzyme for the biosynthesis of L-dopa, offering potential applications in pharmaceuticals and bioindustry.

## Materials and methods

The chemicals were acquired from legitimate companies such as Fisher Scientific, Merck, Acros, and Sigma. L-tyrosine, L-ascorbic acid, zinc sulfate heptahydrate, sodium molybdate, and mercuric sulfate were the chemicals employed in this study.

### Culture maintenance

A wild-type strain of *Aspergillus oryzae* (IIB-9) obtained from the Institute of Industrial Biotechnology (IIB), GC University Lahore's microbial culture bank, was maintained on potato dextrose agar (PDA) slants. Under aseptic circumstances, a homogeneous conidial suspension was prepared by transferring 10 ml of sterile 0.05% monoxal O.T (M.O.T) into a 24 h old *A. oryzae* slant. Turbidity and spore count was analyzed using a spectrophotometer (Shimadzu UV–Visible Spectrophotometer PharmaSpec-1700, China) and a hemocytometer slide-bridge under a compound microscope, respectively. The optical density (*A*_*575*_) and spore count of the suspension were determined to be 1.00 and 3 × 10^4^ CFU/ml, respectively.

### Harvesting fungal biomass

Fungal biomass was harvested aseptically in 250 ml Erlenmeyer flasks under submerged fermentation. Vogel’s medium (25 ml, pH 5.5) containing 2 g/l peptone, 1 g/l yeast extract, 30 g/l glucose, 2 g/l ammonium nitrate, 5 g/l potassium di-hydrogen phosphate, 4 g/l ammonium sulfate, 0.2 g/l magnesium sulfate heptahydrate and, 2.5 g/l tri-sodium citrate was prepared^9^. The flasks were cotton-capped and autoclaved (KT-40L, ALP Co. Ltd., Tokyo, Japan) at 121 °C (15 lbs/in^2^) for 15 min. The flasks were seeded aseptically with 1 ml of conidial suspension. For 48 h, all flasks were placed in a shaking incubator (160 rpm) at 30 °C. The fermented broth was filtered with filter paper to get fungal mycelia. The fungal mycelium was washed multiple times with ice-cold distilled water. Blotting sheets were used to make the mycelium 90% moisture-free, and the fungal mycelium was stored at 4 °C till further use.

### In situ aerobic biotransformation reaction

The aerobic biotransformation reactions for L-dopa production from synthetic L-tyrosine were performed using fungal mycelia as an intracellular tyrosine hydroxylase (TH) enzyme. The dispersion of mycelia was accomplished in the reaction mixture by following the method of^13,14^. Twenty-five milliliters of acetate buffer (50 mM, pH 3.5) containing 0.5 mg/ml intact mycelia, 2.5 mg/ml L-tyrosine, and 5 mg/ml L-ascorbic acid were placed in 100 ml conical flasks. The reaction was executed aerobically in a shaking water bath (80 rpm) at 50 °C for 60 min. The samples were centrifuged at 4,000 rpm for 15 min. The enzyme-containing supernatant was kept at 4 °C in the dark.

### Optimization of the TH production process

UV–Vis spectrophotometric analysis was carried out by varying the volume of Vogel’s medium from 25 to 150 ml, the pH of the fermentation medium from 4.5 to 7.0 with an interval of 0.5, incubation period from 24 to 120 h, and the inoculum size from 0.5 to 3 ml, respectively.

### Analytical techniques

The analytical techniques were determined following the procedures laid down by^15,16^.

### Estimation of L-dopa

One milliliter of supernatant from the reaction mixture, 1 ml of 0.5 N HCl, and 1 ml of nitrite molybdate reagent were added to a test tube. Initially, the yellow color appeared^15^, but adding 1 ml NaOH (1N) resulted in a red coloration of the reaction mixture. The distilled water was used to make the volume up to 5 ml. Absorbance was recorded at 545 nm using a spectrophotometer against a control containing distilled water in place of the supernatant. The total L-dopa production was estimated from the Dopa-standard curve (Fig. S1).

$${\text{y}} = 1.9945{\text{x}} + 0.0005$$

$$1.9945{\text{x}} = {\text{y}} - 0.0005$$

$${\text{x}} = {\text{ y}} - \frac{0.0005}{{1.9945}}$$

$${\text{L}} - {\text{Dopa production}} = \frac{x}{5}$$

### Estimation of residual L-tyrosine

One milliliter of the supernatant was mixed with 1 ml of mercuric sulfate reagent. The tubes were placed in a boiling water bath for 10 min. After cooling, 1 ml of nitrite reagent was added and the volume was raised to 5 ml with distilled water. OD was determined at 545 nm using a spectrophotometer against the control^15^. The amount of L-tyrosine was estimated from the standard tyrosine curve (Fig. S2).

$$y = 0.2105x - 0.0008$$

$$0.2105{\text{x}} = {\text{y}} + 0.0008$$

$$x = y + \frac{0.0008}{{0.2105}}$$

$${\text{L}} - {\text{Tyrosine consumption }}\left( {\frac{{{\text{mg}}}}{{{\text{ml}}}}} \right) = 2.5 - \frac{x}{5}$$

### Tyrosine hydroxylase assay

The enzymatic activity was determined by adopting the following procedure. The samples were prepared by adding 200 µl enzyme, 200 µl L-tyrosine solution in sucrose (100 µg/ml), and 2.6 ml sucrose solution (0.25 M) in a test tube^16^. All the test tubes were placed in a static incubator at 30 °C for 10 min. The absorbance was recorded at 475 nm using a UV–Vis spectrophotometer against the blank.

### Enzyme unit

The enzyme activity unit (IU) was defined as the amount of enzyme required to produce 1 μmol min^−1^ dopachrome under reaction conditions^11^.

$${\text{TH activity }}\left( {\frac{{{\text{IU}}}}{{{\text{ml}}}}} \right) = \frac{{{\text{Abs}} \times {\text{L}}\_{\text{Dopa value}} \times {\text{MW}}}}{3}$$

### Determination of protein content

Quantitatively estimated the total protein content in the enzyme supernatant following the method of^17^, using bovine serum albumin (BSA) as a standard (Fig. S3).

### Synthesis of zinc oxide nanocrystals

The ZnO-NPs were fabricated using high-purity zinc sulfate heptahydrate (ZnSO_4_.7H_2_O) and sodium hydroxide (NaOH) pellets. Powdered ZnSO_4_.7H_2_O (2.874 g, 0.01 mol) with the subsequent addition of NaOH pellets (0.8 g, 0.02 mol) was ground for 30 min. The resulting product was washed with deionized water several times to remove the byproduct, Zn (OH)_2_. Adding NaOH aided in the breakdown of Zn (OH)_2_ due to the increase in heating effect. In the final phase, the product was washed with alcohol. The end product was dried in a hot air oven at 80 °C for 2 h^18^. The ZnO-NPs were made using watch glass (WG) and filter paper (FP). In the WG procedure, a watch glass was used to wash the NPs with deionized water and ethanol before drying them on WG in a hot air oven. While FP method used filter paper to wash the NPs with deionized water and ethanol. WG and FP dried nanoparticles were then employed as a matrix for immobilizing the TH enzyme. To assess the effects of different washing and drying approaches on the properties of the resultant ZnO-NPs and, ultimately, on the immobilization of the TH enzyme, two different washing and drying techniques were used in this study.

### Optimization of enzyme immobilization on ZnO-NPs

The effect of different reaction parameters such as enzyme concentration, ZnO nanoparticles concentration and procurement periods were optimized using the one-factor-at-a-time (OFAT) method to determine the best immobilization conditions. Optimal enzyme concentration was obtained by varying the amount of enzyme from 50 to 300 μl on the WG and FP-dried ZnO-NPs. The amount of ZnO-NPs varied from 25 to 150 mg for watchglass-type and 25–150 mg for filter paper-type nanoparticles while taking optimal enzyme concentrations as control. Moreover, the procurement period variations were studied from 5 to 50 min. The optimal of each variable was then confirmed by UV–Vis spectrophotometric analysis.

### Characterization of ZnO-NPs

The following analytical techniques were employed for the characterization of chemically synthesized ZnO-NPs.

### UV/Vis digital spectrophotometer

The synthesized ZnO-NPs were sonicated in isopropanol for 15 min. A UV spectrum was recorded using a UV–Vis spectrophotometer (Cary 60, Agilent Technologies, USA) for photocatalytic estimation of ZnO-NPs underneath the influence of visible light radiation at a wavelength of 200–800 nm^19^.

### X-ray diffraction (XRD)

XRD (D8 Advance, Bruker-Optik, Ettlingen, Germany) analysis of ZnO-NPs was carried out to determine the crystalline nature and the crystallite size of the nanoparticles^20^. XRD enabled with monochromatized Cu Kα-radiation (*λ* = 0.154 nm) was employed within 2*θ*° range of 5–90° using a scan rate 0.05/min to examine the structure and phase components of the prepared ZnO-NPs. The peaks of radiated X-rays were recorded for the qualitative and quantitative analysis of the specimen.

### Fourier transform infrared (FTIR) spectroscopy

FTIR (Spectrum-100, Perkin Elmer, St. Louis, USA) spectrum of refined and dry ZnO-NPs was determined by spreading the sample on a silicon wafer to make a coated film. The slide was analyzed under an FTIR spectroscope, and the spectrum was recorded in the 4000–400/cm range^21^.

### Scanning electron microscopy (SEM)

To determine the particle size and surface morphology of ZnO-NPs using SEM, they were placed on a carbon-plated platinum strip. The splash drops were cleaned, and the sample was dried in a mercury light for 5 min^22^. A fine electron beam scanned the sample. Electrons interacting with NPs produced different signals on a cathode ray tube and were imaged in SEM.

### Statistical analysis

Treatment effects were compared by post-hoc and protected least significant difference methods under one-way ANOVA (Spss-20) following the method of^23^. The significant difference among the three parallel replicates has been presented, as Duncan’s multiple ranges in the form of probability < *ρ* > values**.**

#### Evaluation of stability and catalytic efficiency of immobilized tyrosine hydroxylase

To optimize the stability of the immobilized enzyme, different times and temperatures were explored, ensuring a successful immobilization process. For this purpose, the immobilized enzyme was incubated at various time intervals (5–45 min) and incubation temperatures (20–60 °C) to determine its stability and thermostability, respectively. The enzyme’s catalytic efficiency was also determined by incubating the immobilized enzyme with different metal salts (Cu^+2^, Fe^+2^, Fe^+3^, Mn^+2^, Zn^+2^). The concentrations of (CuSO_4_.5H_2_O and CuSO_4._7H_2_O) and (FeSO_4_ and FeSO_4_NH_4_) were varied from 10 to 60 mM and 2 to 12 mM, respectively. The effect of Mn^+2^ salts [MnSO_4_, MnO_2_] and Zn^+2^ salts [Zn_3_(PO_4_)_2_, Zn(CH_3_CO_2_)_2_] on immobilized enzyme was evaluated by varying concentrations from 0.5 to 3 Mm for each Mn^+2^ and Zn^+2^ salt. (Note: The optimal outcome of a preceding experiment served as the control for the next experiment).

## Results and discussion

### Production of tyrosine hydroxylase from mycelia of *A. oryzae*

**Figure 2:**
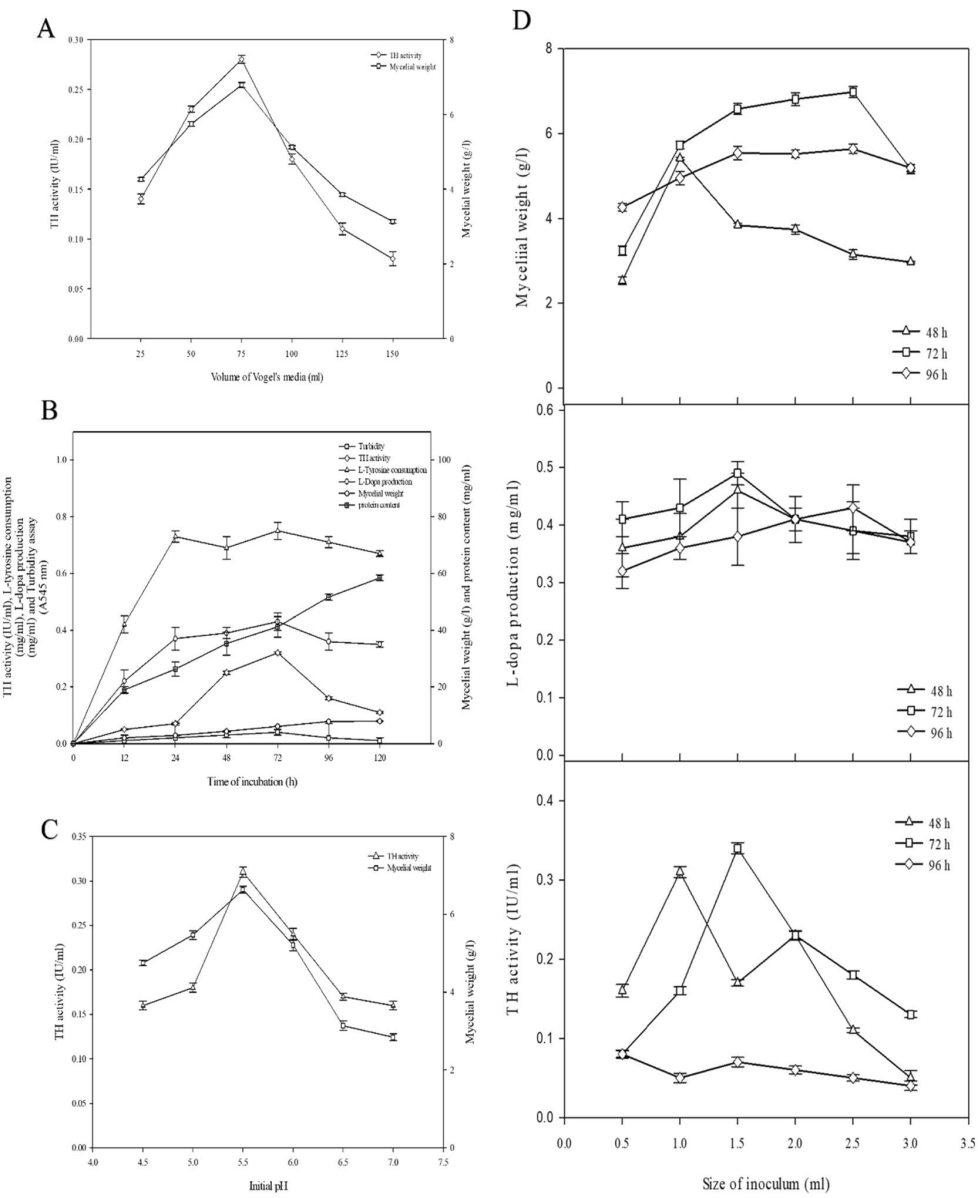
(**A**) Effect of different volumes of Vogel’s medium on the production of TH from mycelia of *Aspergillus oryzae* IIB-9 in 250 ml Erlenmeyer flasks. (**B**) Effect of different times of incubation on the production of TH from mycelia of *Aspergillus oryzae* IIB-9 in 250 ml Erlenmeyer flasks. (**C**) Effect of different initial pH values on the production of TH from mycelia of *Aspergillus oryzae* IIB-9. (**D**) Effect of different sizes of inoculum at different incubation periods on the production of TH from mycelia of *Aspergillus oryzae* IIB-9 in 250 ml Erlenmeyer flasks.

The effect of different volumes of Vogel’s medium on the production of tyrosine hydroxylase from mycelia of *Aspergillus oryzae* IIB-9 was shown in Fig. 2A. At 25 ml volume of media, the TH activity was found to be 0.14 ± 0.06 IU/ml. The highest TH value, i.e., 0.28 ± 0.06 IU/ml, was obtained at 75 ml of media with a mycelial weight of 6.78 ± 0.18 g/l. The increase in the volume of Vogel’s medium resulted in a great availability of nutrients for fungal growth^24^. However after a certain medium volume, more nutrient availability produced more secondary toxic metabolites, leading to a 3.5-fold decrease in the activity of TH detected at 150 ml volume of media. The results of the current study were in contrast with the study of Danial et al*.*^25^, in which they observed maximum enzyme production at a 100 ml volume of media^25^. Although a maximum TH value, i.e., 0.28 ± 0.06 IU/ml was obtained at 75 ml of media. This very low activity is because TH is an intracellular enzyme, which means that it is normally produced inside cells and may not be released into the extracellular environment in large enough quantities. As a result, lower concentrations of intracellular enzymes are frequently found during assaying than for enzymes that are secreted or released naturally. This optimal 75 ml Vogel's medium was then employed for the optimization of the rest of the three reaction parameters for the production of the TH enzyme.

The effect of different times of incubation on the production of TH was examined, as shown in Fig. 2B. The highest TH activity observed at the 72-h incubation period was 0.32 ± 0.04 IU/ml with 0.03 ± 0.01 turbidity, 6.11 ± 0.11 g/l mycelial weight, 0.75 ± 0.03 mg/ml L-tyrosine consumption, 0.43 ± 0.05 mg/ml L-dopa production, and 41.21 ± 3.63 mg/ml protein content. A further increase in the incubation period caused the exhaustion of nutrients in the medium. At 120 h of the incubation period, secondary metabolites interfered with the production of the enzyme^26^. The fungal culture reached the decline phase at 120 h of incubation period, and the activity of TH declined by 65.6% compared to the activity of TH at 72 h incubation period. Our results were in line with the results of^27^, who reported maximum tyrosinase activity (1095 U/mg) at the incubation period of 72 h.

The effect of different initial pH values (4.5–7) on the production of TH from fungal mycelia was studied, as shown in Fig. 2C. TH activity obtained at an initial pH of 4.5 was 0.16 ± 0.05 IU/ml. An increase in TH activity was observed as the pH value shifted toward a neutral level. A maximum increase in the TH activity was detected, i.e., 0.31 ± 0.06 IU/ml at pH 5.5 with a mycelial weight of 6.63 ± 0.09 g/l. The decline in TH activity beyond pH 5.5 was due to the denaturation of the enzyme and altered microenvironment, thus disturbing the ideal conditions required for the optimum activity of TH enzyme. The results of the current study were in contrast with the results of^28^, as they observed maximum tyrosinase activity from *Agaricus bisporus* at optimum pH 7. TH activity was also examined by^16^, in which the maximum TH activity was reported at pH 5.8.

The effect of the different sizes of inoculum at three different incubation periods (48 h, 72 h, and 96 h) on TH production was determined, as shown in Fig. 2D. The increase in the inoculum size from 0.5 to 1.5 ml at the 72 h incubation period caused a 4.2-fold increase in the activity of TH. Further increase in the inoculum size caused a decrease in the activity of TH. At 96 h of incubation, maximum TH activity was found with 2.5 ml inoculum size, i.e., 0.33 ± 0.04 IU/ml. The TH activity and L-dopa production at 72 h incubation period with 1.5 ml inoculum size was 0.34 ± 0.07 IU/ml and 0.49 ± 0.09 mg/ml L-dopa production, respectively. The results of the present study were not in line with the results of Krishnaveni et al.^26^, as they reported maximum tyrosinase activity with the inoculum size of 2.5 ml by using *Acremonium rutilum*^27^*.* Haq et al.^28^ reported the highest yield of L-dopa (1.28 mg/ml) using the mutant strain of *A. oryzae* UV-1 with an inoculum size of 4 ml/100 ml^29^.

### Enzyme immobilization on ZnO nanocrystals

**Figure 3:**
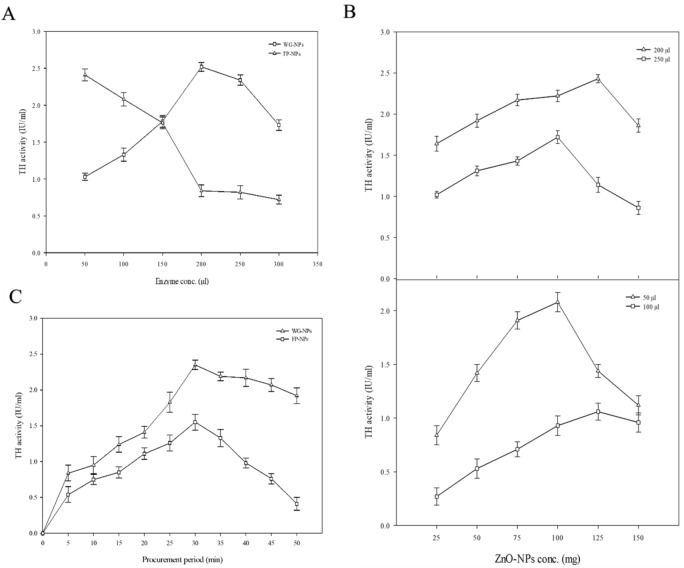
(**A**) Effect of enzyme concentrations for its immobilization on ZnO-NPs. (**B**) Effect of different ZnO-NPs concentrations for its immobilization on TH. C Effect of different procurement periods for the immobilization of TH on ZnO-NPs.

Different enzyme concentrations were adsorbed on the WG and FP nanoparticles, and TH activity was determined for each level as shown in Fig. 3A. The TH activity with 50 µl enzyme concentrations using WG-NPs was 1.03 ± 0.05 IU/ml. At 200 µl enzyme concentration, maximum TH activity was obtained with WG-NPs, i.e., 2.52 ± 0.14 IU/ml. The maximum TH activity with FP-NPs was observed at an initial 50 µl enzyme concentration, i.e., 2.41 ± 0.08 IU/ml. The maximum TH activity observed with WG-NPs at 200 µl enzyme concentration was 1.04-fold higher than the TH activity observed with FP-NPs at 50 µl enzyme concentration. The increase in enzyme loading directly leads to enhancing the activity of an enzyme. However a high amount of enzyme loading can impede the reaction^30^. Due to this, enzyme molecules might develop protein–protein interactions. Dogac et al*.*^30^ reported maximum enzyme activity on iron oxide magnetic NPs with 1 mg/ml enzyme concentration^31^.

The effect of different ZnO-NPs concentrations on two different enzyme concentrations for WG-NPs (200 and 250 µl) and two different enzyme concentrations for FP-NPs (50 and 100 µl) was studied. TH activity was determined for each level, as shown in Fig. 3B. The maximum TH activity of 125 mg WG-NPs with 200 µl enzyme concentration was 14% higher than the TH activity of 100 mg FP-NPs with 50 µl enzyme concentration. The low amounts of NPs may not provide enough carriers for the immobilization of the enzyme. Dogac et al*.*^30^ reported optimum enzyme activity on iron oxide magnetic NPs with 25 mg concentration^31^. Husain et al*.*^31^ observed maximum enzyme activity obtained from *A. oryzae* by varying concentrations of ZnO-NPs (25–150 mg) and obtained maximum enzyme activity at 100 mg ZnO-NPs^32^.

The effect of different procurement periods (5–50 min) for the immobilization of TH on ZnO-NPs was observed, as shown in Fig. 3C. The maximum TH activity (2.35 ± 0.06 IU/ml) and L-dopa production (0.63 ± 0.07 mg/ml) with WG-NPs at 30 min procurement period was 2.7-fold higher than the TH activity at 5 min procurement period. Similarly, the maximum TH activity and L-dopa production with FP-NPs was recorded at 30 min procurement period, i.e., 1.55 ± 0.11 IU/ml and 0.57 ± 0.09 mg/ml. The maximum TH activity of WG-NPs was 34% higher than the TH activity of FP-NPs, while L-dopa with WG-NPs was 1.10-fold higher than FP-NPs during 30 min procurement period. The increase in the procurement period may provide more time for binding the enzyme with NPs. A further increase in the procurement period above the optimum point may cause a hindrance in enzyme activity^33^. Moreover, Dogac et al.^30^ reported the maximum enzyme production with iron oxide NPs at 15 min procurement period^32^.

### Characterization of ZnO nanocrystals

**Figure 4:**
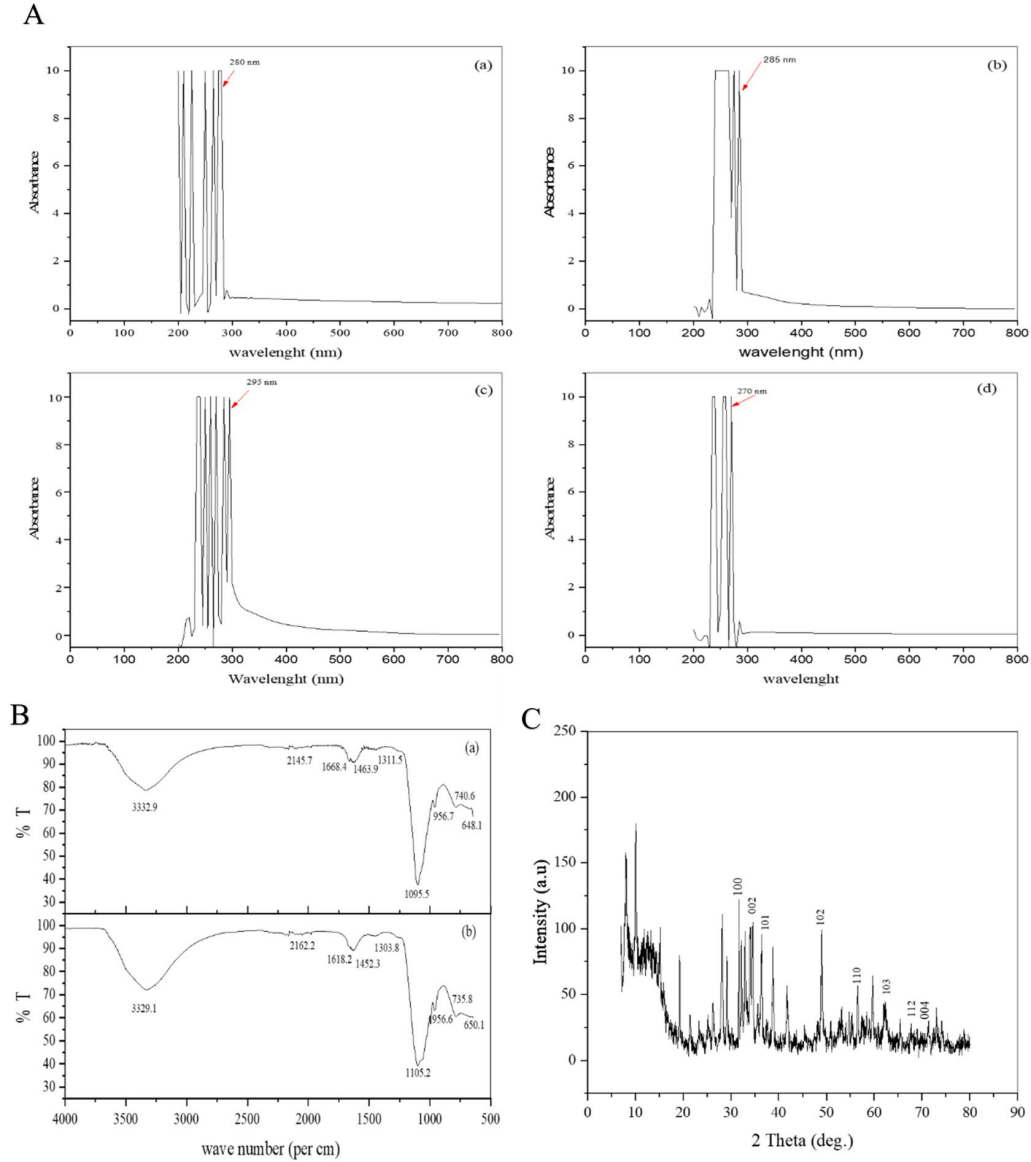
(**A**) UV–Visible absorption spectrum of ZnO-NPs synthesized by solid-state. (**B**) FTIR spectra of ZnO-NPs synthesized by solid state reaction. (**C**) XRD patterns of WG-ZnO-NPs synthesized by solid state reaction.

The homogeneously dispersed two types of ZnO-NPs (WG-NPs and (FP-NPs) were analyzed at different wavelengths (200–800 nm) by dispersing them separately in deionized water and isopropanol, as shown in Fig. 4A. The WG-ZnO-NPs gave maximum absorption at 280 and 295 nm by dispersing them in deionized water and isopropanol, respectively. The FP-ZnO-NPs gave maximum absorption spectra by dispersing them in deionized water and isopropanol at 270 and 285 nm, respectively. The absorption spectrum of WG-ZnO-NPs in isopropanol was 1.03 times greater than that observed when dispersing FP-ZnO-NPs in isopropanol. Awodugba et al*.*^33^ reported excitonic peaks of ZnO-NPs at 277 and 235 nm^34^.

FTIR spectrophotometric analysis was performed to detect the presence of stabilizing functional groups adsorbed on the crystal surface of chemically synthesized ZnO-NPs, as shown in Fig. 4B. In the FTIR spectrum of WG-ZnO-NPs and FP-ZnO-NPs, a strong absorption band was observed at 3332.9 and 3329.1/cm, respectively, which may be due to O–H hydroxyl functional group. The absorption bands at 2145.7 and 2162.2/cm of WG-ZnO-NPs and FP-ZnO-NPs were due to the stretching vibrations of C–H linked groups. The bands at 1311.5 and 1303 cm^−1^ of WG-ZnO-NPs and FP-ZnO-NPs corresponded to C = O asymmetric stretching vibration. The absorption band at 1668.4 and 1618.2/cm of WG-ZnO-NPs and FP-ZnO-NPs, respectively were due to the C = O symmetric stretching vibration. The bands at 1095.5 and 1105.2/cm of WG-ZnO-NPs and FP-ZnO-NPs, respectively corresponded to C–O stretching vibrations. The observed band from 740/cm to 648.1/cm and 735.8/cm to 650.1/cm showed the stretching vibrations of WG-ZnO-NPs and FP-ZnO-NPs, respectively. Our results were closely related to the findings of Jayarambabu et al*.*^34^, who studied the absorption band of ZnO-NPs at 3457.5, 2853.4, 1634.6, 1322.1, 1075, and 731.9/cm due to the stretching vibrations of O–H, C–H, C = O, C–O, and C–H groups. The slight difference in values might be due to distinct approaches for synthesizing ZnO-NPs^35^.

The XRD patterns of ZnO-NPs shown in Fig. 4C determined the peak intensity, position, and width data of NPs. The sharp intensity of peaks indicated that ZnO nanocrystals were formed. The peaks located at 31.64° (100), 34.56° (002), 36.28° (101), 48.92° (102), 56.5° (110), 62.16° (103), 67.68° (112), and 71.24° (004) confirmed the crystalline nature of ZnO-NPs. The intensity peaks observed at 32.92°, 33.99°, 38.88°, 41.72° and 59.72° showed the presence of impurities, while other small peaks indicated background noise. The Powder X software cataloged the ZnO-NPs peaks (JPCDS card number: 36–1451). The results of the current study almost followed the results of Jayarambabu et al*.*^34^, in which the researcher’s reported intensity of peaks for ZnO-NPs at 31.7° (100), 34.5° (002), 36.2° (101), 47.7° (102), 56.5° (110), 62.2° (103) and 68.4° (112)^35^. Talam et al.^35^ also reported nearly the same results in their research work^36^.

**Figure 5:**
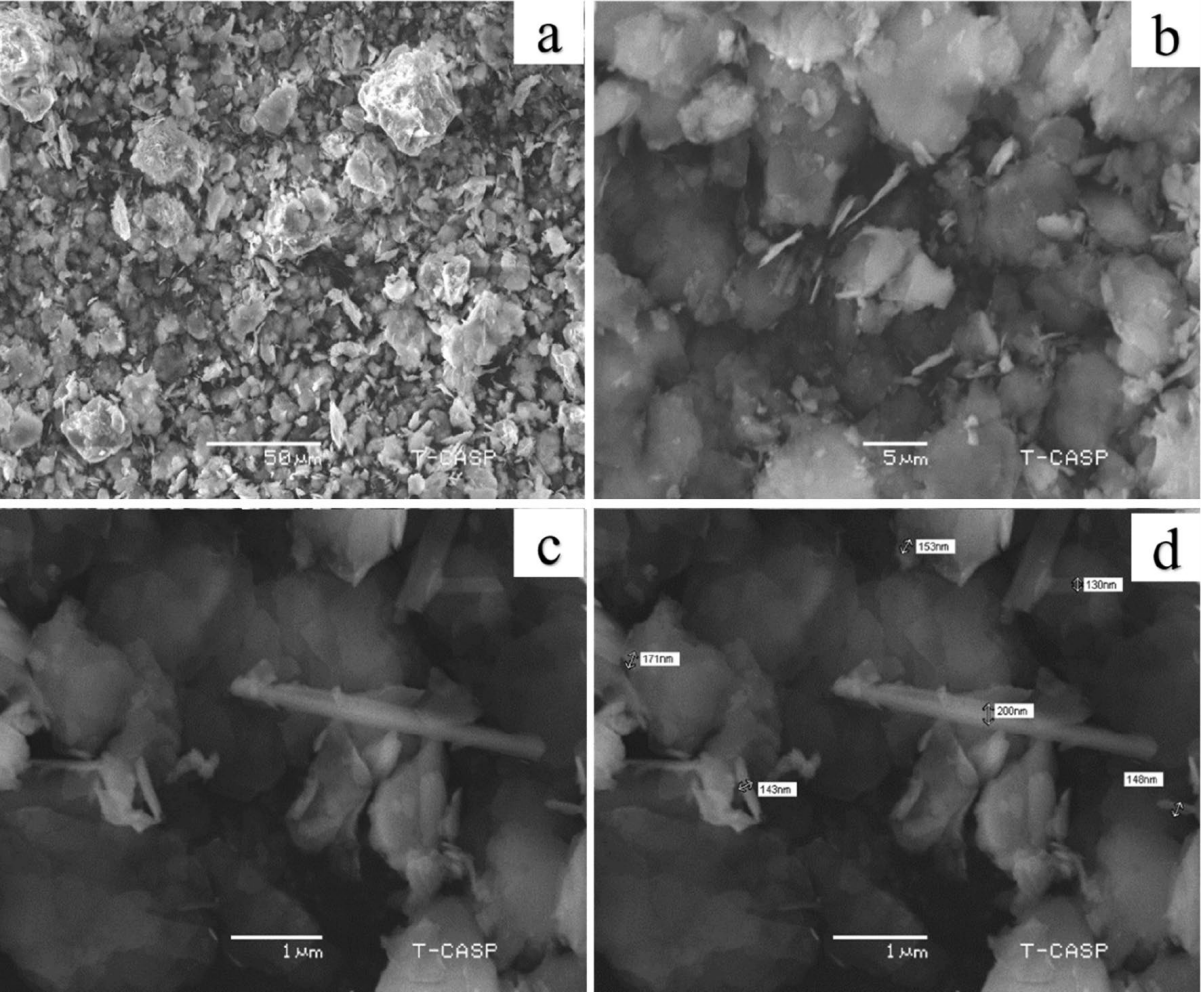
SEM micrographs of WG-ZnO-NPs resolved at different magnifications (50, 5, and 1 µm).

Figure 5 revealed the SEM micrographs of ZnO-NPs, which were resolved at different magnifications, i.e., 50, 5 and 1 µm. These images confirmed the formation of the irregularly shape clusters of ZnO-NPs. The particle size of these ZnO-NPs was between 130 and 170 nm. Large-sized ZnO nanocrystals were obtained in this study, to maximize binding sites for TH immobilization, thus facilitating nano-bioconjugate formation and stable aggregates. However, our results were contradict the finfings of Hutera et al*.*^36^, who described the particle size of ZnO-NPs in the 40–60 nm range^37^.

### Improved stability and catalytic efficiency of immobilized tyrosine hydroxylase

**Figure 6:**
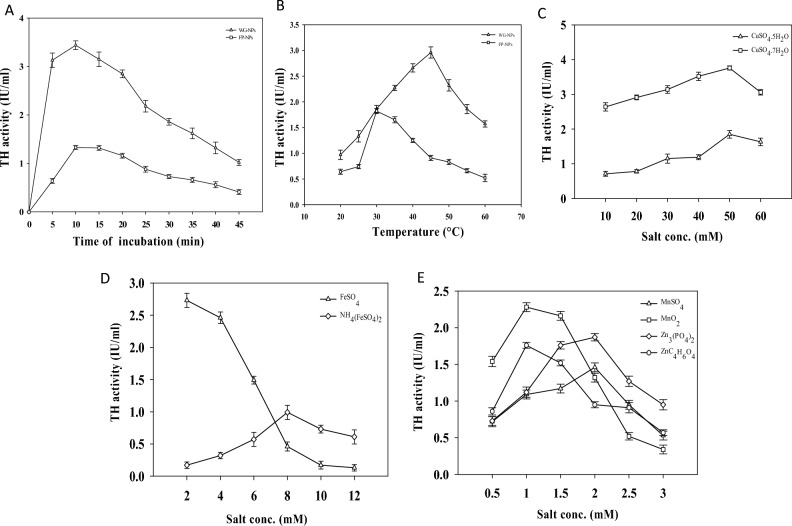
(**A**) Analysis of TH stability over different time of incubation for its immobilization on ZnO-NPs. (**B**) Analysis of TH stability over different temperatures for its immobilization on ZnO-NPs. (**C**) Comparative analysis of different copper salts for the improved TH catalytic efficiency immobilized on ZnO-NPs. (**D**) Comparative analysis of different iron salts for improved TH catalytic efficiency immobilized on ZnO-NPs. (**E**) Comparative analysis of different manganese and zinc salts for the improved TH catalytic efficiency immobilized on ZnO-NPs.

The effect of different incubation times (5–45 min) on immobilized TH was studied to improve its stability, as shown in Fig. 6A. The maximum TH activity was obtained at 10 min incubation time with WG-NPs, i.e., 3.44 ± 0.09 IU/ml. A 3.37-fold decrease in the activity of TH was found at 45 min with WG-NPs. The maximum TH activity with FP-NPs was obtained at 15 min, i.e., 1.32 ± 0.05 IU/ml. The highest TH activity recorded with WG-NPs at 10 min was 61% more than the maximum TH activity found with FP-NPs at 15 min. According to Arsalan et al*.*^37^, a longer incubation period for an enzyme reaction result in more product formation, but the relationship is not linear^38^. However, our results were aligned with the findings of Duarte et al*.*^38^, in which they reported maximum tyrosinase stability at 15 min of incubation^39^.

The effect of different temperatures (20–60 °C) on immobilized TH to improve its stability was studied, as shown in Fig. 6B. A threefold rise in TH activity with WG-NPs, i.e., 2.96 ± 0.11 IU/ml, was observed at 45 °C. The maximum TH activity obtained with FP-NPs at 30 °C was 1.82 ± 0.05 IU/ml. The highest TH activity with WG-NPs at 45 °C was 38% higher than the TH activity obtained with FP-NPs at 30 °C. The decrease in TH activity at elevated temperatures beyond the enzyme's optimal range, can lead to enzyme denaturation, causing the enzyme to lose its native conformation and loss of catalytic function. Tan et al. reported the thermostability of immobilized tyrosinase on polyhydroxyalkanoate (PHA) nano-granules at 55 °C^40^.

The effect of two copper salts, CuSO_4_.5H_2_O and CuSO_4_.7H_2_O, with different concentrations (10–60 mM) on immobilized TH to improve its catalytic efficiency was shown in Fig. 6C. At 50 mM salt concentration of CuSO_4_.5H_2_O, a maximum of 1.85 ± 0.11 IU/ml TH activity was recorded. The optimal TH activity recorded with CuSO_4_.7H_2_O was at 50 mM salt concentration, i.e., 3.76 ± 0.06 IU/ml. The highest activity of TH recorded with 50 mM CuSO_4_.7H_2_O was 51% higher than that recorded with 50 mM CuSO_4_.5H_2_O. Tyrosinase, a copper-containing enzyme, can utilize CuSO_4_ to enhance enzyme activity as a stimulator^41^. The lower concentration of CuSO_4_ enhanced the dopa production. Surwase et al*.*^41^ reported that 0.06 mg/ml concentration of CuSO_4_ enhanced L-dopa production by 0.468 mg/ml^42^. Tan et al. checked the effect of copper ions in different concentrations (0–10 mM) on immobilized tyrosinase in polyhydroxyalkanoate (PHA) nano-granules and reported maximum enzyme activity at low concentrations of copper ions (0.01–1 Μm)^40^.

The effect of two iron salts, FeSO_4_ and NH_4_(FeSO_4_)_2_, with different salt concentrations (2–12 mM) on TH-immobilized ZnO-NPs was examined, as shown in Fig. 6D. At 2 mM FeSO_4_ concentration, a maximum of 2.73 ± 0.11 IU/ml TH activity was recorded. With the further increase in the FeSO_4_ salt concentration, the TH activity declined 21-fold, as observed at 12 mM FeSO_4_ salt concentration. The maximum TH activity found with NH_4_(FeSO_4_) was at 8 mM, i.e., 0.99 ± 0.11 IU/ml. The highest TH activity with 2 mM FeSO_4_ was 63% higher than the TH activity recorded with 8 mM NH_4_(FeSO_4_). Tyrosinase is an iron-binding enzyme, and iron acts as a cofactor for enzyme activity^43–45^. The ferrous ion is required to oxidize various monohydric phenols to dihydric phenols under the action of tyrosinases. The 0.002 mg/ml concentration of ferrous salt can affect the activity of tyrosinases^46^. The findings of the current study were according to the results of Petrack et al*.*, in which the researchers reported ferrous ions as slight activator for the tyrosinase activity and produced L-dopa 6.4 mol/min and ferric ions as an inhibitor for the tyrosinase activity^47^.

The four different salts (MnSO_4_, MnO_2_, Zn_3_(PO_4_)_2_, and ZnC_4_H_6_O_4_) with different concentrations (0.5–3 mM) were analyzed, and the activity of TH was determined for each salt concentration, as shown in Fig. 6E. The maximum TH activity with MnSO_4_ was recorded at 1.5 mM salt concentration, i.e., 1.17 ± 0.09 IU/ml. A further increase in the MnSO_4_ concentration resulted in a decrease in the TH activity. The maximum TH activity with MnO_2_ was recorded at 1 mM salt concentration, i.e., 2.28 ± 0.09 IU/ml. It is 1.9-fold more than the TH activity recorded at 1.5 mM MnSO_4_. The maximum TH activity recorded with Zn_3_(PO_4_)_2_ was at 2 mM salt concentration, i.e., 1.87 ± 0.05 IU/ml, and the optimal TH activity recorded with ZnC_4_H_6_O_4_ was at 1 mM salt concentration, i.e., 1.76 ± 0.04 IU/ml. The highest TH activity with 2 mM Zn_3_(PO_4_)_2_ was 5.8% more than the TH activity recorded with 2 mM Zn_3_(PO_4_)_2_. The present study did not follow the results of Yamamoto et al*.*, in which they reported manganese salt as a stimulator for the activity of TH and increased the activation of the enzyme by 136%^48^. However, our results were similar to those of Xiao et al.^49^, in which they checked the effect of zinc ions (0–30 µM) on the iron-binding TH enzyme^49,50^. In a nutshell, this study is promising because immobilization of TH on ZnO-NPs resulted in 8.7-fold increase in TH activity as compared to the pSiNPs and many other nanosupports that have no significant impact on TH activity, either due to the conformational changes or the blockage or partial coverage of the catalytic site^51–54^. Similarly, TH immobilization into the lipid core of maltodextrin nanoparticles didn’t cause any increase in the activity and stability of TH enzyme^55^. Hence, this study indicates the true potential of ZnO-NPs in improving the stability and catalytic efficiency of TH enzyme after immobilization process.

## Conclusions

For TH production from *A. oryzae*, different reaction parameters, like the volume of Vogel's medium, the time of incubation, the initial pH, and the size of the inoculum were optimized, resulting in an overall 17.6% increase in the activity of TH. ZnO-NPs were fabricated via the chemical method to improve the biocatalyst’s stability and catalytic efficiency resulting in 1.28-fold increase in L-dopa production after immobilization. The SEM images confirmed the particle size of these ZnO-NPs between 130 and 170 nm. The maximum TH catalytic efficiency obtained by immobilizing it on ZnO-NPs was 3.76 ± 0.06 IU/ml. Immobilization of TH on ZnO-NPs resulted in an 8.7-fold increase in TH activity. The effect of different salts, i.e., copper, iron, manganese, and zinc on immobilized TH, were investigated to increase the catalytic efficiency. The immobilized TH can improve L-dopa production to cure Parkinson’s disease. Further, scale-up studies are required to improve the activity of TH for the production of L-dopa.

### Supplementary Information

Supplementary Figures.

## Data Availability

All data generated in this research work has been included in the manuscript.
