# Supplementary material for: Immobilization of Aspergillus oryzae tyrosine hydroxylase on ZnO nanocrystals for improved stability and catalytic efficiency towards L-dopa production
Source: Sci Rep. 2023 Dec 18;13:22882. doi: 10.1038/s41598-023-50198-x (PMC10739923; doi:10.1038/s41598-023-50198-x)
Supplement: Supplementary file 1 — Supplementary Figures. [file 41598_2023_50198_MOESM1_ESM.docx]

**Supporting information**

**Fig. S1** Standard curve of L-tyrosine

**Fig. S2** Standard curve of L-dopa

**Fig. S3** Standard curve of BSA
